# Impact of ketogenesis and strong ion difference on acid-base in our CICU

**DOI:** 10.1186/cc10753

**Published:** 2012-03-20

**Authors:** T Clark, B McGrath, P Murphy, M Jayarajah

**Affiliations:** 1 Derriford Hospital, Plymouth, UK

## Introduction

Persistence of a mild metabolic acidosis or base deficit was occasionally observed in our otherwise well patients post cardiac surgery, sometimes delaying discharge. We hypothesised that this metabolic abnormality may be due to either ketogenesis caused by a combination of starvation and the surgical stress response, or strong ion imbalances following fluid administration. The administration of large volumes of chloride-rich fluids (as may occur during cardiac surgery to prime the cardiopulmonary bypass circuit or resuscitate the patient) is known to induce hyperchloraemic metabolic acidosis [1]. Using simplifications of the original Fencl-Stewart's equations, it is possible to partition the base deficit into its constituent parts, subsequently determining the relative contribution of chloride, albumin and unmeasured anions to acidosis [2,3]. Ketone production may contribute significantly to the unmeasured anion component.

## Methods

A prospective cohort analysis. Fifty postoperative cardiac patients were recruited. For each we measured urinary ketones three times per day for the first 48 hours of their CICU admission. Arterial blood gas (ABG) data were recorded in conjunction each time. For each blood gas we partitioned the base deficit into its constituent components using previously published equations [1-3].

## Results

A total of 231 ABGs were analysed. Urinary ketones were checked along with 181 of the ABGs. A total of 14 ketonuria checks were positive (8%) in 11 patients (22%). In nine ABGs ketonuria was associated with a significant base deficit, whilst in three it was also associated with a metabolic acidosis. The average starvation time was 39 hours (SD 11 hours). In 121 (52%) ABGs the chloride component of the base deficit (BECl) was below -2. In 104 (45%) ABGs the BECl contributed to greater than 75% of the BETOTAL, whilst in 74 (32%) of these the BECl was greater than the BETOTAL. In 18 ABGs a BECl of less than -2 caused a metabolic acidosis.

## Conclusion

Our observation of persistent metabolic abnormalities in otherwise well postoperative cardiac patients may be due to iatrogenic strong ion imbalances caused by hyperchloraemic solutions. Ketogenesis was not a significant contributing factor. The impact of relative hyperchloraemia on pH was buffered by other counteracting metabolic factors (for example, hypoalbuminaemia), as in 74 ABGs the BECl was greater than the BETOTAL.
